# THE DEMENTIA CARE PARTNER HUB: A NOVEL RESOURCE TO SUPPORT PEOPLE LIVING WITH DEMENTIA, CARE PARTNERS, AND CARE TEAMS

**DOI:** 10.1093/geroni/igae098.1931

**Published:** 2024-12-31

**Authors:** Walter Dawson, Jenn Hollandsworth Reed, Sherril Gelmon

**Affiliations:** Oregon Health & Science University, Portland, Oregon, United States; Portland State University, Portland, Oregon, United States; Portland State University, Portland, Oregon, United States

## Abstract

Care partners of people living with dementia (PLWD) need information and access to multiple services and programs to provide support and maintain their own wellbeing. Focus groups (n=5) and interviews (n=24) were conducted with care partners and interviews with organizations serving PLWDs in the Portland, Oregon region between July 2022 and September 2023. A review of organizational websites and relevant literature identified several US-based role model programs. Data analysis revealed community assets and unmet needs, and articulated resource priorities to inform programs/services. Dementia care partners need multiple supports for caregiving: information about dementia; navigation and coordination; psychological support; recognition of care partner burden; leisure activities; day services; transportation; legal and financial services; home health; and financial support. Feedback revealed care partner deficits in accessing information and social supports. To augment existing services and supports for care partners, a Dementia Care Partner Hub (the “Hub”) should be developed. The Hub would be the central node of a regional network of existing services. The Hub should serve all dementia care partners in its service region, with a responsive design to the specific assets and needs of historically and currently underrepresented communities. It should augment and complement current clinical, research, and social programs by connecting care partners to programs and services. Community-specific information for communities defined by geography and identity will ensure the Hub equitably serves care partners with varying needs. Key elements to achieving this equity goal include language access, culturally appropriate information, and accessibility of the Hub across the entire region.

